# Hedgehog Dermatophytosis: Understanding *Trichophyton erinacei* Infection in Pet Hedgehogs and Its Implications for Human Health

**DOI:** 10.3390/jof9121132

**Published:** 2023-11-24

**Authors:** Lucia Kottferová, Ladislav Molnár, Peter Major, Edina Sesztáková, Katarína Kuzyšinová, Vladimír Vrabec, Jana Kottferová

**Affiliations:** 1 Clinic of Birds, Exotic and Free Living Animals, University of Veterinary Medicine and Pharmacy, Komenského 73, 04181 Košice, Slovakia; lucia.kottferova@uvlf.sk (L.K.); ladislav.molnar@uvlf.sk (L.M.); edina.sesztakova@uvlf.sk (E.S.); katarina.kuzysinova@uvlf.sk (K.K.); vladimir.vrabec@uvlf.sk (V.V.); 2 Department of Public Veterinary Medicine and Animal Welfare, University of Veterinary Medicine and Pharmacy, Komenského 73, 04181 Košice, Slovakia; jana.kottferova@uvlf.sk

**Keywords:** hedgehog, infections, *Trichophyton erinacei*, zoonoses, mycoses, dermatophytoses

## Abstract

Pet hedgehogs, which are increasingly favoured companions, have garnered attention due to their potential as carriers of zoonotic diseases. These small insectivorous mammals, native to Europe, Asia, and Africa, are commonly kept as pets. The encroachment of humans into hedgehog habitats has brought these animals closer to people, raising concerns about disease transmission. This article reviews the current knowledge regarding zoonotic disease associated with pet hedgehogs, with a particular focus on mycotic infections caused by *Trichophyton erinacei*. Data from various regions and hedgehog species are synthesised to assess the significance of pet hedgehogs as potential reservoirs and transmitters of zoonotic pathogens. Our study highlights the importance of understanding the health risks associated with pet hedgehogs and underscores the need for continued research to mitigate zoonotic disease transmission from these potentially disease-carrying companions.

## 1. Introduction

In recent years, the upsurge in exotic pet ownership has introduced a charming yet intricate dimension to the human‒animal bond. Among unconventional companions, pet hedgehogs have garnered considerable attention. However, sharing our lives with these animals has not been without challenges, particularly concerning the transmission of fungal infections. *Trichophyton erinacei*, a dermatophytic fungal pathogen, has garnered scientific interest in recent years. Its potential to serve as a zoonotic bridge between these unconventional pets and their human caretakers has raised significant concerns within the veterinary and public health communities. A thorough scientific investigation is necessary to fully understand the complex landscape of zoonotic potential and public health consequences surrounding *T. erinacei* transmission from pet hedgehogs. Hedgehogs, small nocturnal insectivorous mammals widespread across Europe, Asia, and Africa, comprise sixteen recognised species organised into five genera: *Hemiechinus* (two species), *Atelerix* (four species), *Erinaceus* (four species), *Paraechinus* (four species), and Mesechinus (two species) [1,2,3,4].

Dermatomycosis infections from *T. erinacei* primarily originate from imported, unprotected white-bellied or African pygmy hedgehogs (*Atelerix albiventris*) available for purchase in pet shops, as well as Egyptian long-eared hedgehogs (*Hemiechinus auritus*). Understanding and addressing the potential health implications associated with these hedgehog species in the context of exotic pet ownership are crucial [5].

This paper is a literature review of the zoonotic pathogen *Trichophyton erinacei* in domestic hedgehogs, which is important from scientific and practical points of view, especially for veterinarians and owners of domestic hedgehogs. This article aims to shed light on the multifaceted aspects of this fungal pathogen’s transmission dynamics, clinical manifestations, and preventive strategies. Ultimately, it seeks to inform both hedgehog owners and healthcare professionals to mitigate the risks associated with these unique animal companions while fostering harmonious coexistence.

## 2. Classification and Taxonomy of the Dermatophytes

Dermatophytes, a group of highly successful pathogenic fungi, are responsible for causing superficial mycoses, commonly referred to as dermatophytosis or ringworm, in both humans and animals. These fungi are taxonomically and ecologically related, belonging to the family *Arthrodermataceae* within the order *Onygenales*. What sets them apart is their unique capability to metabolise keratin as their exclusive nutrient source [6,7].

Traditionally, dermatophytes were classified into three genera: *Trichophyton*, *Epidermophyton*, and *Microsporum*, with their sexual states grouped under *Arthroderma* [8,9]. However, recent taxonomic revisions, the elimination of dual nomenclature, and the utilisation of multi-gene phylogenies have expanded the number of genera within the dermatophyte group. Despite these changes, many of the most clinically significant primary pathogenic species still fall within the original three genera [10]. In the newly proposed taxonomy, *Trichophyton* contains 16 species, namely *Epidermophyton* (1 species), *Nannizzia* (9 species), *Microsporum* (3 species), *Lophophyton* (1 species), *Arthroderma* (21 species), and *Ctenomyces* (1 species), but more detailed studies remain needed to establish species borderlines. Each species now has a single valid name. Two new genera have been introduced: *Guarromyces* and *Paraphyton*. The number of genera has increased, but species that are relevant to routine diagnostics now belong to smaller groups, which enhances their identification [8].

One notable taxonomic refinement involves the former entity known as *T. mentagrophytes *var.* erinacei*. Smith and Marples first described it in 1963, but it has since undergone a reclassification and is now officially known as *Trichophyton erinacei* [9]. This reclassification aligns with earlier observations by mycologists who referred to it as the “hedgehog mushroom” [11,12]. The recognition of *T. erinacei* as a distinct dermatophyte species dates back to 1966 when Quaife emphasised that it is not a mere variant or subspecies but indeed constitutes a separate species [13].

In the last few decades, dermatophytes have come a long way in their modern systematic classification, which is mostly based on molecular species identification from ITS sequences [13]. The molecular investigations suggest a close genetic or evolutionary relationship between *T. erinacei* and the *Trichophyton anamorph* of *A. benhamiae*. This relationship extends to the teleomorph *A. benhamiae*. The findings indicate that this close relationship is observed in strains of these fungi found in both the American‒European and African regions [14,15].

Throughout the years, the aetiological agent of ringworm in hedgehogs has been reported under different names, reflecting evolving species concepts. These names include *T. erinacei *var.* erinacei*, *T. erinacei*, *T. mentagrophytes *var.* erinacei*, *Arthroderma benhamiae *var.* erinacei*, and *A. benhamiae*. These taxonomic refinements reflect a comprehensive understanding of the fungal species and their intricate relationships [12,16].

## 3. Clinical Signs

### 3.1. Clinical Signs in Hedgehogs

**Figure 1:**
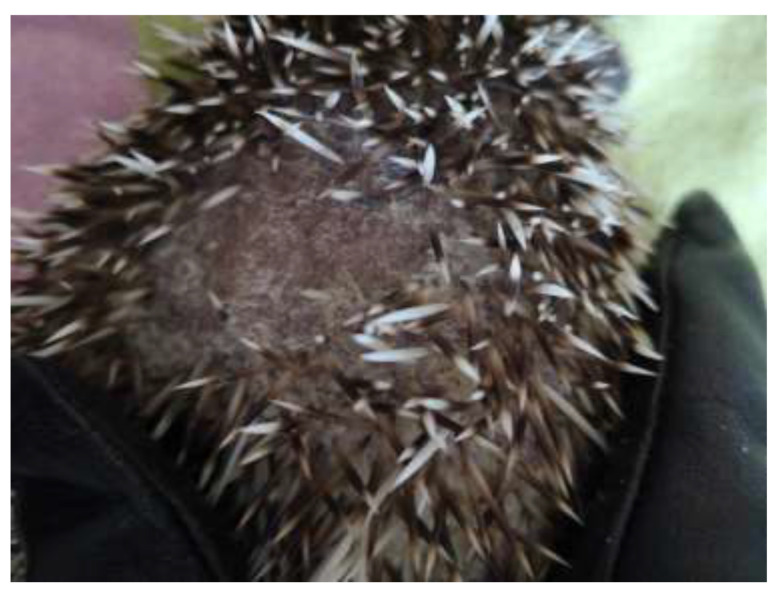
Crusty lesions, alopecia, and loss of spines in pet hedgehog caused by mycotic infection.

**Figure 2:**
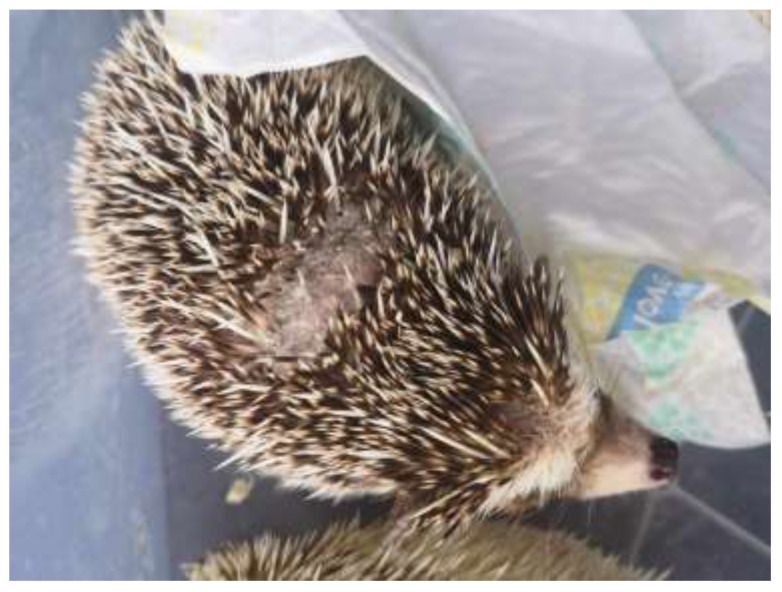
Lesion on the back of an African pygmy hedgehog due to infection by *T. erinaceus*.

*T. erinacei* infections in hedgehogs are characterised by several clinical signs. These signs are essential for recognising and understanding the disease in these animals. Hedgehogs infected with *T. erinacei* often exhibit crusty lesions on their skin [17]. These lesions can appear as scaly patches or areas of skin with an abnormal texture. Additionally, infected hedgehogs may experience alopecia, which is the loss of fur or spines, as you can see in Figure 1 and Figure 2. These clinical signs are particularly prominent on the head area of the hedgehog or on their back [8,18].

It is important to note that the course of *T. erinacei* infection in hedgehogs can vary. While some individuals may display noticeable clinical signs, others may experience only mild or even asymptomatic infections. This variability underscores the complexity of the disease in hedgehogs [2,8,19,20].

### 3.2. Clinical Signs in Humans

*Trichophyton* infections in humans often present with distinctive clinical signs, primarily affecting the skin. One of the most common initial signs is the emergence of itchy and inflamed skin eruptions, often displaying a reddish base at the point where the skin comes into contact with an infected animal. While these symptoms can start off as mild, they tend to intensify over time. Unfortunately, there are instances where the initial rash resembles other inflammatory skin conditions, such as eczema, leading to the improper use of topical or oral corticosteroids. Regrettably, this misdiagnosis can expedite the progression of the infection, making it more severe [21].

Usually, there are single lesions in humans, but several cases have had two or three separate body areas affected. When evaluating skin lesions, particularly on the hands, obtaining a thorough and accurate medical history is crucial. This history should include any recent contact with animals, particularly hedgehogs. In cases where recent animal contact is reported, especially with hedgehogs, maintaining a high level of suspicion is vital for initiating prompt and appropriate treatment. Early recognition of *Trichophyton* infection and the commencement of antifungal therapy can lead to the timely relief of symptoms and clearance of the infection [22,23,24].

Furthermore, *T. erinacei* infections in humans can manifest in various ways (Table 1), primarily affecting the skin. Recognising these clinical signs is pivotal for accurate diagnosis and tailored treatment [2]. In human cases, *T. erinacei* infections frequently result in inflammatory dermatological conditions, with two common presentations being *tinea manus* and *tinea corporis*. These infections can cause discomfort and skin abnormalities, including redness, itching, and, in some instances, blister formation [18].

However, *T. erinacei* does not stop there; it has been identified as the causative agent in a range of other dermatophyte infections, including *Tinea faciei*, *Tinea capitis*, and *Tinea barbae* [21,22]. These diverse clinical manifestations highlight the critical need for precise species identification and treatments tailored to the specific infection [24,25,26].

**Table 1:** Clinical presentation in humans after contact with hedgehog infected by *T. erinaceus*.

Clinical Signs in Humans	Region	Authors
*Tinea corporis*	Asia	[22,27,28]
	Europe	[29,30]
*Tinea faciei*	America	[31]
	Asia	[27,32]
	Europe	[28,33,34]
*Tinea barbae*	Europe	[25]
*Tinea manus*	America	[21]
	Asia	[26,28,35,36,37]
	Europe	[30,38,39,40,41]
*Onychomycosis*	Asia	[42]

## 4. Transmission

Understanding the transmission of *T. erinacei* from hedgehogs to humans, and both among hedgehogs and among humans, is crucial for preventing and managing infections. This pathogen is classified as an “emerging pathogen” and has been identified globally, with cases reported in various regions, including Chile, North Africa, and Taiwan. The increasing recognition of this pathogen underscores the importance of understanding its transmission dynamics [24,25]. *T. erinacei* exhibits a notable propensity for zoonotic transmission, primarily from hedgehogs to humans. Human infections often result from direct contact with infected hedgehogs or their environments. The presence of spines covering the hedgehog’s body increases the risk of transmission when handled without gloves. They could potentially cause injuries that might increase the risk of infection. If a hedgehog were to scratch or pierce the skin of another animal or a human, it could create a pathway for pathogens to enter the body, leading to an increased risk of infection. [24,26,27,36,38,42,43].

The prevalence of *T. erinacei* infection in hedgehog populations varies by region. Studies in New Zealand and Spain have reported infection rates ranging from 30% to nearly 45% among examined hedgehogs [12,44,45]. In a study by Smith and Marples [45], they looked at 114 European hedgehogs living in New Zealand suburbs. The researchers collected materials from the hedgehogs’ hair and spines and cultured them on Sabouraud’s agar plates. They observed that *T. erinacei* strains grew in 44.7% of the 115 samples. They provided insights into the prevalence of British hedgehogs. Furthermore, according to them, the prevalence of *T. erinacei* infection in British hedgehogs was estimated to be around 30% [44]. In a separate investigation, Ruszowski [46] looked into the presence of bacterial, viral, protozoan, and fungal pathogens in hedgehogs, including both wild and domesticated pet hedgehogs. The research encompassed the identification of zoonotic pathogens among hedgehogs, regardless of their health status, and whether they were clinically healthy, sick, or deceased. Hedgehogs should be regarded as an important component of the epidemiology of various zoonotic infections due to the prevalence of numerous pathogens within them, including zoonotic ones, and their close proximity to humans.

In addition to direct contact, environmental factors play a role in transmission. *T. erinacei* has been isolated from bedding material, such as sawdust, where infected hedgehogs reside. This highlights the potential for indirect cross-infection among hedgehogs and other species, including humans [43]. Smith and Marples [45] came to the conclusion that *Caparinia ripilis* was the primary cause of most hedgehog scabbiness, with *T. erinacei* frequently recoverable from such lesions. They also suggested that mites could potentially transmit ringworm between animals, but most new infections were attributed to contact between infected mother hedgehogs and their offspring.

There have been documented cases of transmission from infected mothers to their offspring. This vertical transmission further highlights the potential for the persistence and spread of the fungus within hedgehog populations [38]. Research has indicated that hedgehog mites may serve as potential vectors for *T. erinacei* transmission between hedgehogs and other species, including humans [31].

In a study conducted in Spain [12], researchers examined 20 pet hedgehogs suspected of suffering from dermatophytosis. Their investigation involved both microbiological and molecular examinations, which revealed that 50% of the animals tested positive for *T. erinacei*. This suggests that *T. erinacei* infections are a significant concern with hedgehogs, with varying prevalence rates in different regions. 

## 5. Identification of *T. erinacei*

The challenge in distinguishing *T. erinacei* from other dermatophytes is rooted in their morphological resemblance. This underscores the vital importance of meticulously gathering a comprehensive patient history, especially regarding interactions with hedgehogs. When inflammatory dermatomycosis follows hedgehog contact or injury, it becomes paramount to conclusively confirm the presence of *T. erinacei* [31].

The conventional differentiation of *T. erinacei* presents challenges due to the absence of species-specific macro- and micromorphological features. The fast-growing, white, radiating thallus with a partly granular surface is not unique to *T. erinacei*; it can be seen in other dermatophyte species as well. Additionally, the bright yellow pigmentation observed on the colony reverse is not consistently present and can vary with culture conditions [39]. Microscopically, *T. erinacei* is characterised by cylindrical and club-shaped, multi-septate macroconidia, along with elongated and round microconidia that insert at right angles to the hyphae. The density and arrangement of microconidia can vary [43]. The urea cleavage test, historically used for *T. erinacei* identification, has yielded mixed results, making it an unreliable criterion [47].

Only molecular DNA-based tests, which typically involve sequencing, can identify isolated dermatophytes in humans. These tests are what confirm the identity of species. Molecular biological methods stand out as the definitive means for distinguishing *T. erinacei* from dermatophyte species that share similar morphological characteristics. The sequencing of the ITS region of rDNA and the TEF-1α gene region has proven to be highly effective in achieving crystal-clear differentiation between *T. erinacei* and its closely related counterparts. This remarkable distinction holds its ground even when dealing with *T. erinacei* strains originating from diverse hedgehog sources [12,48,49].

Diagnostic laboratories have been the first to use cutting-edge molecular techniques to deal with these problems, notably matrix-assisted laser desorption/ionisation time-of-flight mass spectrometry (MALDI-TOF MS) for the identification of dermatophytes. These advanced methods hold the promise of significantly improving the accuracy and efficiency of species identification, potentially providing a solution to the discrepancies that have often plagued conventional approaches [50].

In light of these advancements, it is becoming clearer that more research into *T. erinacei* strain typing and differentiation is not only needed but also essential for fully comprehending this new pathogen [51,52]. From a veterinary perspective, the increasing trend in exotic pet ownership, including hedgehogs, underscores the pressing need for heightened vigilance among veterinary professionals. Given the dynamic nature of zoonotic dermatophytosis linked to these pets, it is imperative to be informed of the most recent developments in medical diagnosis. The advent of these molecular identification methods has significantly enhanced our ability to swiftly and precisely identify *T. erinacei* and other dermatophytes in hedgehogs. This not only aids in the prompt treatment of affected animals but also mitigates the risk of zoonotic transmission to humans [41,48,51,53].

## 6. Therapy

### 6.1. Therapy in Hedgehogs

Dermatophyte infection presents a diverse range of clinical manifestations, which can make it difficult to diagnose and treat the infection effectively [9,26].

Although topical medications are frequently utilised, their application is complicated by the hedgehog’s thick, multidirectional spines and its defensive balling-up manoeuvre. Washing hedgehogs can be challenging, and stressful for the animal, and also increases the danger of contracting a zoonotic disease for the handler. Washes containing lime sulphur or enilconazole can be effective against dermatophytes. Only when the animal is under anaesthesia may some bodily parts, such as the head and ventrum, be accessible, which raises the danger of fluid inhalation and hypothermia [10,16,20].

Systemic therapy is generally more effective and convenient and reduces the need for additional animal handling. Griseofulvin has previously been used to treat dermatophytosis in hedgehogs but is no longer recommended due to concerns about safety and efficacy [5,54,55,56,57]. The recommended dosage of itraconazole for the treatment of dermatophytosis is 5–10 mg/kg once or twice daily as reported for the African pygmy hedgehog (*Atalerix albiventris*) [58]. The dose calculation in the study by Bexton et al. [56] used the current trial for hedgehogs, yielding a dose of 10 mg/kg twice daily. In the same study, as terbinafine has a wide margin of safety and higher doses are generally regarded as being necessary to treat dermatophytosis, a relatively high dose of 1.0 mg/kcal in hedgehogs was selected [59]. The dosages used for both drugs for hedgehogs in this study appeared to be safe and effective. Hedgehogs in this study demonstrated no adverse effects with either drug. Both drugs were administered with food, which may have reduced the risk of gastrointestinal disturbance. Both drug treatments were effective for the management of hedgehogs infected with dermatophytosis. However, terbinafine was more effective at eliminating fungi from the skin and hair, resulting in a mycological cure after 14 days of therapy of 92.8% of hedgehogs compared with 36.6% of hedgehogs treated with itraconazole [55].

The treatment of hedgehog dermatophytosis should involve a combination of topical and systemic antifungal agents [31]. Topical antifungal agents with activity against dermatophytes, including azoles, ciclopirox olamine, amorolfin, and tolnaftate, can be considered as part of the treatment regimen [19,53]. It is essential to choose the most appropriate treatment based on the individual case and the response to therapy [31]. Recommended treatment can include a combination of topical enilconazole and systemic antifungal therapy with itraconazole, or ketoconazole [9,19,53]. Topical terbinafine has also demonstrated effectiveness as a standalone treatment for hedgehog dermatophytosis, and it can be used in combination with oral therapy [12]. It is important to note that when dermatophytosis is diagnosed in one hedgehog, all other hedgehogs in the group should also be treated to prevent further spread [26,54].

The identification of terbinafine-resistant strains is clinically significant when *T. erinaceus* is isolated from hedgehogs. There have already been reports of allylamine resistance in Asian and European nations [60,61,62]. Additionally, the frequency of clinical isolates that were resistant to terbinafine varied from less than 1% in Switzerland [32] to more than 70% in India [63]. According to the literature, India is one of the Asian countries with the highest prevalence of resistant dermatophytes. The European countries of Poland [64,65], Finland [60], Denmark [66], Switzerland [27], and Russia [61] are among those where dermatophyte isolates that are resistant to conventional treatment are still present. Therefore, a crucial task for medical mycologists is the detection of drug-resistant strains, animal species affected, disease states, and the indication of their geographic range.

### 6.2. Therapy in Humans

The most common diseases in humans are those caused by dermatophytes. The current arsenal of antifungal medications consists of topical and systemic medications, the latter of which are largely used to treat infections of the hair and nails [67,68]. The latter pose a significant therapeutic challenge because treatment may be time-consuming and may have unfavourable effects, especially in the vulnerable elderly population [67,69]. Systemic terbinafine or itraconazole must be used for one to twelve weeks in order to treat severe lesions (hyperkeratotic, inflammatory, and folliculitis) [17,70,71]. Imidazoles, allylamines, and morpholines are effective topical treatments for minor conditions, in pregnant or nursing women, and in circumstances with substantial oral drug interactions. The cure rate is around 80%. Topical therapy is additionally administered as an adjuvant during oral therapy and to stop recurrence following systemic therapy [12,72,73].

In Mexico, Lammoglia et al. reported the case of a 31-year-old male with tinea incognito and Majocchi’s granuloma due to *T. erinacei* from a hedgehog, treated successfully with terbinafine 250 mg P.O. daily for 6 weeks [74]. Alejandra et al. [75] studied in vitro antifungal susceptibility for *T. erinacei*. In this study, a high MIC for amphotericin B (4 µg/mL) and a resistance level for fluconazole (64 µg/mL) were found. The patient in this study was successfully treated with terbinafine (250 mg/day), a drug that showed high in vitro susceptibility. A clinical case of a woman who appeared with pruritic erythema on the right palm was described by Takashi Mochizuki et al. [76] The erythema had been treated with topical steroids for four weeks, but it had continued to spread. The erythema was hyperaemic and infiltrated irregularly. Its centre showed no indications of healing, its perimeter had few vesicles, and its edge had scales. Later, the erythema was diagnosed as tinea manuum and was treated with topical terbinafine once daily. After a few days of treatment, the erythema flared up and red papular erythemas formed on the skin on the back of both hands and on both forearms. Topical terbinafine was continued for the palm, and fluocinolone acetonide ointment was administered to the newly developed lesions on the arms. After 4 weeks of treatment, all of the inflammatory eruptions had receded. Lysková et al. [29] presented five case reports of tinea faciei and tinea corporis skin fungal infections caused by the zoophilic species *T. erinacei* in four young women and a boy. All patients had white-bellied hedgehogs (*Atelerix albiventris*) at home and all agreed that the hedgehogs had no visible clinical signs of infection. These are the Czech Republic’s first detailed documented cases. The first patient’s infection on the face was treated locally with ciclopiroxolamine, which resulted in regression of the symptoms. The therapy was then supplemented with systemically administered terbinafine, and she was successfully cured. Three patients presented with forearm lesions that were successfully treated in two cases with systemically administered terbinafine and the use of ciclopiroxolamine on a local level. The fifth patient, who had a flank lesion, was first treated with a combination of topical ciclopiroxolamine and fusidic acid and was later cured after the infection reappeared after the application of econazole cream. This therapy was effective in every case. Successful therapy with terbinafine has also been described in the literature [31,40]. Successful therapy with griseofulvin, ketoconazole, econazole, clotrimazole, and itraconazole is described in the literature, and various combinations of these and other preparations are often mentioned [27,30,37]. However, cases of failure of topical clotrimazole monotherapy are also reported [36], and a case of failure of therapy with systemically administered griseofulvin in combination with cream containing clotrimazole [27] has been reported. The therapy of skin foci caused by *T. erinacei* is usually quite lengthy, lasting several weeks to months [17,27,35,36,70,76].

## 7. Prevention

When it comes to ensuring the health and well-being of pets, as well as humans, prevention is of the utmost significance. In the context of health, the old saying, “an ounce of prevention is worth a pound of cure”, is especially true. We feel that the preventive approach, emphasising the importance of thorough screening in pet stores and post-purchase veterinary consultations, is critical. We can minimise the chances of adopting pets with infectious diseases or other health issues by undertaking comprehensive screening in pet stores. Post-purchase visits to the vet clinic, on the other hand, serve as an important line of defence, allowing professionals to examine the pet’s overall health, address any underlying health issues, and provide advice for responsible ownership. Furthermore, when it comes to pet health, our article emphasises the need for early detection. The clinical indications of diseases are not always clear, which is why regular vet appointments should be a usual practice for pet owners. Furthermore, when skin diseases are suspected, fungal culture can be quite beneficial. We can avoid more serious and costly health problems by diagnosing and treating any skin diseases as soon as they arise. A proactive approach to prevention is not simply a choice in a society where our pets are not just animals but essential parts of our families; it is a responsibility.

## 8. Conclusions

In the realm of mycology, *T. erinacei* has garnered recognition as a significant zoophilic dermatophyte. The growing trend of keeping exotic animals as pets has ushered in a new frontier in dermatology practice. This surge in exotic pet ownership has led to the emergence of several zoonotic diseases that can potentially be transmitted to humans. These zoonotic concerns underscore the need for heightened awareness and recognition within the field of dermatology. As dermatologists encounter an expanding array of dermatophyte infections linked to exotic pets, a comprehensive understanding of these conditions and their management becomes imperative. In this dynamic environment, where the human‒animal bond extends to these exotic pets, veterinarians play a pivotal role in safeguarding both the health of these animals and the well-being of their owners. By staying informed, adopting advanced diagnostic techniques, and fostering collaboration with dermatologists and other healthcare professionals, veterinarians can contribute to a safer and healthier coexistence between humans and their pet companions.

## Data Availability

Not applicable.
